# Inhibitor of p52 NF-κB subunit and androgen receptor (AR) interaction reduces growth of human prostate cancer cells by abrogating nuclear translocation of p52 and phosphorylated AR^ser81^

**DOI:** 10.18632/genesandcancer.77

**Published:** 2015-09

**Authors:** Farideh Mehraein-Ghomi, Dawn R. Church, Cynthia L. Schreiber, Ashley M. Weichmann, Hirak S. Basu, George Wilding

**Affiliations:** ^1^ University of Wisconsin Carbone Cancer Center, Madison, Wisconsin, USA; ^2^ University of Texas MD Anderson Cancer Center, Houston, TX, USA

**Keywords:** NF-κB2/p52, AR, protein-protein interaction inhibitor, prostate cancer

## Abstract

Accumulating evidence shows that androgen receptor (AR) activation and signaling plays a key role in growth and progression in all stages of prostate cancer, even under low androgen levels or in the absence of androgen in the castration-resistant prostate cancer. Sustained activation of AR under androgen-deprived conditions may be due to its interaction with co-activators, such as p52 NF-κB subunit, and/or an increase in its stability by phosphorylation that delays its degradation. Here we identified a specific inhibitor of AR/p52 interaction, AR/p52-02, via a high throughput screen based on the reconstitution of *Gaussia* Luciferase. We found that AR/p52-02 markedly inhibited growth of both castration-resistant C4-2 (IC_50_ ∼6 μM) and parental androgen-dependent LNCaP (IC_50_ ∼4 μM) human prostate cancer cells under low androgen conditions. Growth inhibition was associated with significantly reduced nuclear p52 levels and DNA binding activity, as well as decreased phosphorylation of AR at serine 81, increased AR ubiquitination, and decreased AR transcriptional activity as indicated by decreased prostate-specific antigen (PSA) mRNA levels in both cell lines. AR/p52-02 also caused a reduction in levels of p21^WAF/CIP1^, which is a direct AR targeted gene in that its expression correlates with androgen stimulation and mitogenic proliferation in prostate cancer under physiologic levels of androgen, likely by disrupting the AR signaling axis. The reduced level of cyclinD1 reported previously for this compound may be due to the reduction in nuclear presence and activity of p52, which directly regulates cyclinD1 expression, as well as the reduction in p21^WAF/CIP1^, since p21^WAF/CIP1^ is reported to stabilize nuclear cyclinD1 in prostate cancer. Overall, the data suggest that specifically inhibiting the interaction of AR with p52 and blocking activity of p52 and pARser81 may be an effective means of reducing castration-resistant prostate cancer cell growth.

## INTRODUCTION

About 30% of all prostate cancer patients after first line of therapy succumb to recurrent prostate cancer. Although the recurrent prostate cancer regresses after androgen deprivation therapy (ADT), the majority of these patients return to the clinic with the refractory disease known as castration-resistant (CR) prostate cancer, for which successful therapy remains a challenge. Accumulative evidence suggests that castration-resistant activation of androgen receptor (AR) and development of apoptosis-resistant cells play key roles in the transition of androgen-dependent prostate cancer to CR prostate cancer [1].

AR is a nuclear receptor that in its inactive form resides in the cytoplasm. Upon binding to its ligand, it undergoes various modifications that facilitate its translocation to the nucleus, where it performs its transcriptional effect either by itself or by interacting with its co-activators [2]. Among the post-translational modifications, phosphorylation plays an important role in AR activity [3]. The majority of phosphorylation sites are mapped in the N-terminal domain (NTD), which also contains the transactivation domain of AR [4,5]. Only a few phosphorylation sites have been reported at the Ligand Binding Domain (LBD) and DNA Binding Domain (DBD) of AR [4]. The phosphorylation sites and the kind of kinases involved determine most of the AR activities, e.g., transactivation, nuclear translocation, ubiquitination and finally its degradation [3-5]. It has been reported that phosphorylation at ser81 (pAR^ser81^) occurring 6 to 8 hours after androgen stimulation plays an important role in AR transcriptional activity [4,6]. Other reports indicate the importance of phosphorylation of AR at ser81 for its stability, e.g., inhibiting its ubiquitination and subsequent proteasomal degradation [6-8].

Castration-resistant AR activation and signaling under very low androgen levels or in the absence of androgen may be due to a variety of mechanisms that alter the sensitivity and/or specificity of AR activation. These mechanisms include: AR gene amplification leading to increased sensitivity to low levels of androgen, mutations in AR gene that alter its response to other steroids and growth factors, expression of splice variants of AR (e.g., AR-V7) that lack the Ligand Binding Domain (LBD) and therefore may be constitutively active in the absence of androgen, increased AR stability by phosphorylation, and aberrant activation of AR by interaction with other co-activators such as p52 NF-κB subunit [9,10].

Most members of the NF-κB family of proteins, which consists of RelA/p65, NF-κB1/p50, c-Rel, RelB, and NF-κB2/p52 (p52 NF-κB subunit), have been shown to be aberrantly activated in prosate cancer cells and tissues [11]. In the canonical pathway of NF-κB activation, heterodimer p65/p50 is constitutively expressed in prostate cancer [12]. In the less explored non-canonical NF-κB2 (p100/p52) pathway, protein p52 induces the expression of genes that are involved in hyperplasia, growth and cell proliferation [13]. In a previous study, we showed that NF-κB DNA binding activity increases after 72 hours in LNCaP human prostate carcinoma cells treated with synthetic androgen R1881 [14]. Subsequently, Lessard *et al*, [15] showed that specifically p52 NF-κB subunit translocates to the nucleus in LNCaP cells treated with R1881 after 72h. Overproduction of p52 has been observed in several solid tumors including prostate cancer [16]. It has been shown that overexpression of p52 induces castration-resistant growth in human prostate carcinoma LNCaP cell xenografts by inhibiting both cell cycle arrest and apoptotic cell death induced by androgen deprivation [17]. Nadiminty *et al*, [10] showed that p52 induces castration-resistant growth in LNCaP cells by causing an aberrant activation of AR in the androgen-independent condition. Recently, it was also shown that the resistance of prostate cancer cells to the next-generation antiandrogen, enzalutamide, is due to increased expression of p52, which is mediated by aberrant AR activation and AR splice variant production [18]. The reciprocal regulation of p52 and AR splice variants, such as AR-V7, has been proposed as a possible mechanism of the resistance to enzalutamide [18]. In our recent publication [19], we proposed an autocrine feed forward loop involving SSAT enzyme, reactive oxygen species (ROS) and NF-κB that may sustain ROS production and p52 activation in low androgen environment in prostate cancer cells, contributing to castration-resistant prostate cancer progression. Based on these studies, we hypothesize that inhibiting the interaction of AR and p52 may prevent the castration-resistant growth and enzalutamide resistance of prostate cancer cells.

Here, using a *Gaussia* Luciferase (GL) reconstitution assay [20], we firmly established that AR interacts directly with p52 under androgen-deprived conditions. We used this GL reconstitution method in a high throughput screen (HTS) on 2,800 small molecules in a Life Chemicals Library [21] to identify four drug-like small molecules that specifically inhibited the AR/p52 protein-protein interaction. As none of the four inhibitors competed with androgen for binding to the AR LBD in a competition assay, they were classified as non-antiandrogens, which is important for our goal of specifically blocking non-androgen activation of AR. The compounds were further characterized for cell growth inhibitory effects in two human prostate cancer cell models: androgen-dependent LNCaP and its castration-resistant variant C4-2 cell lines [22]. Based on growth inhibitory activity as well as ability to decrease AR transcriptional activity, we selected one compound, AR/p52-02, for further studies on mode of action including effect of the compound at growth inhibitory doses on p52 and AR nuclear levels, phosphorylation/stability of AR, and p21^WAF1/CIP1^ levels.

Although the assumed role of p21^WAF1/CIP1^ is regulating the cell cycle by inhibiting the cell cycle kinases [23], there are reports that show the association of p21^WAF1/CIP1^ with castration-resistant growth of prostate cancer [24, 25]. In patients who relapsed after ADT, the level of p21^WAF1/CIP1^ is even higher than seen before castration [26, 27]. This points to the association of high p21^WAF1/CIP1^ expression with advanced prostate cancer [28], which is considered an unexpected outcome, as p21^WAF/CIP1^ is regarded as an anti-proliferative factor [23]. Other reports further emphasized the role of p21^WAF/CIP1^ as a direct AR target gene, in that its expression correlates with androgen stimulation and mitogenic proliferation in prostate cancer [28-30].

Mode of action studies showed that AR/p52-02, at growth inhibitory doses, caused decreases in nuclear p52 levels and pAR^ser81^ as well as decreased AR stability. Interestingly, we found that AR/p52-02 reduces p21^WAF1/CIP1^ level in both LNCaP and C4-2 cells only in the *presence* of androgen. Overall, the results of this study indicate that small molecule inhibitor of the interaction of AR and p52 NF-κB subunit, AR/p52-02, represses castration-resistant prostate cancer cell growth by blocking both AR and p52 pathways, and shows promise for development of a new therapeutic agent for castration-resistant prostate cancer.

## RESULTS

### Expression of vector containing fusion of p52 NF-κB subunit with C-terminal domain of *Gaussia* Luciferase and establishment of AR/p52 interaction via *Gaussia* Luciferase reconstitution assay

**Figure 1:**
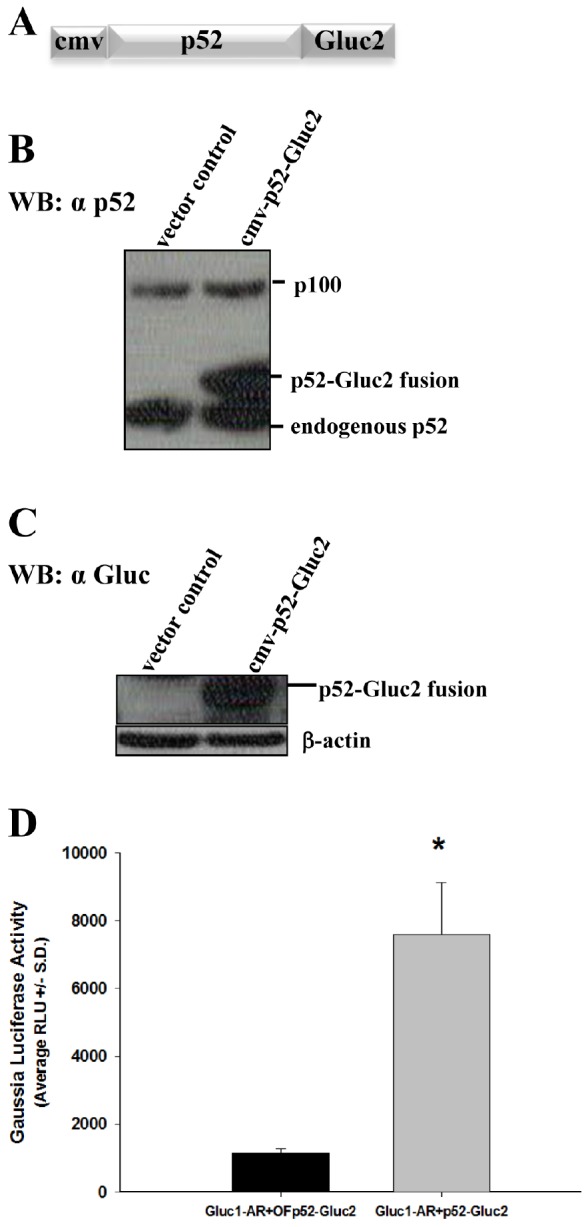
The vector containing fusion gene NF-κB2/p52-Gluc2 expresses the fusion protein, and Gaussia Luciferase is reconstituted due to p52 and androgen receptor interaction Hep3B cells were transfected with vector control or the vector containing the fusion gene cmv-p52-Gluc2 (A). Cell lysates were collected after transfection and analyzed by western blot (WB) using antibody for NF-κB2/p52 (B) or antibody for *Gaussia* Luciferase (Gluc) (C). In (D), *Gaussia* Luciferase activity was measured in the lysate from Hep3B cells co-transfected with cmv-Gluc1-AR^≠^ + cmv-p52-Gluc2 or cmv-Gluc1-AR + cmv-OF-p52-Gluc2 (out-of-frame fusion of p52 with GLuc2; control vector). A significant 6-fold greater activity (* *P* < 0.0002) was observed in cells co-transfected with cmv-Gluc1-AR+cmv-p52-Gluc2 when compared to cmv-Gluc1-AR+cmv-OFp52-Gluc2 control, indicating expression of the p52-Gluc2 fusion protein as well as reconstitution of *Gaussia* Luciferase due to interaction of AR and p52. N=6 per condition for *Gaussia* Luciferase activity studies. ^≠^cmv-Gluc1-AR vector was published previously [31].

For investigating the direct protein-protein interaction between AR and p52, we used the *Gaussia* Luciferase (GL) reconstitution assay [20]. This technique is based on reconstitution of reporter enzyme GL in live cells. The gene encoding for the enzyme was split into two sections: N-terminal section (GLuc1) and C-terminal section (GLuc2). The construction of cmv-GLuc1-AR vector was reported before [31]. Here, we report the construction of vector cmv-p52-GLuc2. The fusion construct that schematically is shown in Figure 1A was transfected into Hep3B cells and the cell lysate was probed either with a monoclonal antibody for p52 at the N-terminal of the fusion protein (Figure 1B) or polyclonal antibody against GL at the C-terminal of the fusion protein (Figure 1C). Fusion protein p52-Gluc2 has a higher molecular weight compared to that of the endogenous p52 (Figure 1B). The in-frame fusion of p52 with Gluc2 yielded a band that is not present in cell lysate from cells transfected with vector control (Figure 1C). These data confirmed the expression of the p52-Gluc2 fusion protein and lack of endogenous GL in Hep3B cells. As shown in Figure 1D, co-transfection of Hep3B cells with the cmv-p52-Gluc2 vector and our previously published cmv-Gluc1-AR vector [31] yielded a significant 6-fold increase in GL bioluminescence activity compared to control (*P*<0.0002), demonstrating reconstitution of GL due to the interaction of AR protein with p52 protein at 48 hours after transfection of the fusion vectors, and thus firmly establishing AR/p52 interaction.

### Identification of specific inhibitors of AR/p52 interaction

**Figure 2:**
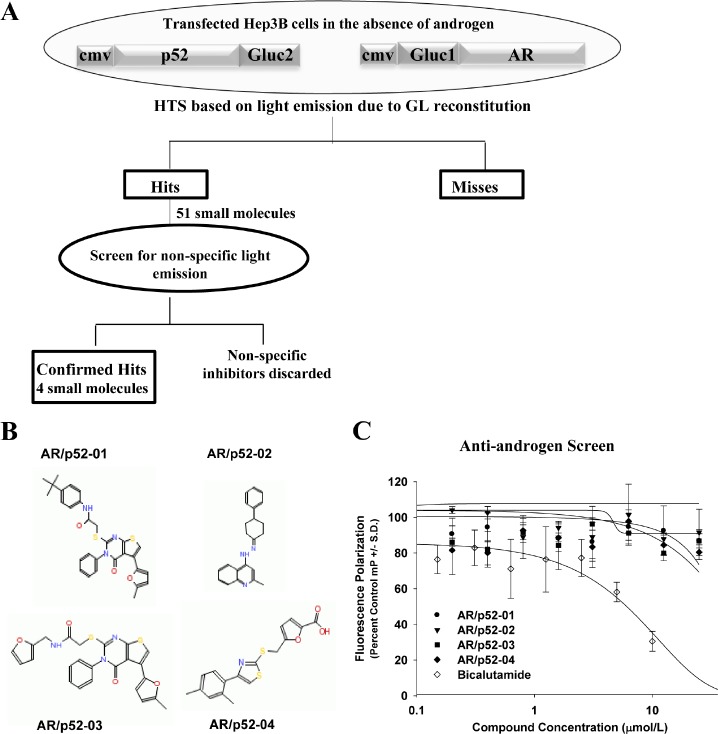
Screening for inhibitors of p52 NF-κB subunit and AR interaction A high throughput screening assay based on *Gaussia* Luciferase reconstitution in cell lysate from Hep3B cells that were co-transfected with vectors cmv-p52-Gluc2 and cmv-Gluc1-AR was performed on a Life Chemicals Library subset containing 2,800 small molecules, as shown in (A). Four ‘hits’ were discovered that specifically inhibited the direct interaction of p52 with androgen receptor (AR) in the absence of androgen. Structures of the four small molecule inhibitors of AR/p52 interaction are shown in (B). The four inhibitors were further screened using the PolarScreen™ Androgen Receptor Competitor Assay to eliminate any with antiandrogenic properties (C). Bicalutamide (Casodex®), a clinical antiandrogen, was used as a control. A classic dose response of bicalutamide competition with fluorescence tagged androgen (fluoromone) for binding to the ligand binding domain (LBD) of AR was observed. In contrast, none of the AR/p52 inhibitors competed with androgen for binding to LBD of AR in the same dose range. Based on this assay the compounds were all classified as non-antiandrogens. N=3 per data point, nonlinear regression fit curves, representative from two assays performed for each compound.

The level of bioluminescence from reconstituted GL due to the interaction of AR and p52 proteins was sufficient for utilization in a high throughput screening (HTS) assay to identify specific inhibitors of AR/p52 interaction. A schematic representation of the GL reconstitution-based HTS is shown in Figure 2A. The detailed procedure for HTS using GL reconstition vectors was reported previously [32]. The structures of the four small molecule inhibitors of AR/p52 interaction found by the HTS are shown in Figure 2B, and Table 1 shows the Life Chemicals Library identification numbers for the compounds. Since the goal was to identify inhibitors that specifically block non-androgen activation of AR, i.e., inhibitors that block the activation of AR by co-activators that do not interact with the ligand binding domain (LBD) of AR, the compounds were further screened to eliminate any that competed with androgen for binding to the AR-LBD. As shown in Figure 2C, while clinical antiandrogen bicalutamide showed a classical dose response competition with androgen for binding to the AR-LBD, none of the AR/p52 inhibitors competed with androgen in the same dose range. The inhibitors were thus classified as non-antiandrogens.

**Table 1:** Small molecule inhibitors of AR/p52 and their ∼IC50 values for inhibition of growth of LNCaP or C4-2 cells in the absence or presence of androgen

Life Chemicals^a^ ID	HTS^b^ Hit ID	−Rc		+R^c^	
		LNCaP	C4-2	LNCaP	C4-2
F1174-2988	AR/p52-01	3 μM	4 μM	8 μM	>10 μM
F1174-3266	AR/p52-02	4 μM	6 μM	>10 μM	>10 μM
F1441-0714	AR/p52-03	>10 μM	>10 μM	>10 μM	>10 μM
F2135-0668	AR/p52-04	>10 μM	>10 μM	>10 μM	>10 μM

a Small molecules from Life Chemicals Inc.

b HTS: high throughput screen

c R: synthetic androgen R1881

### Inhibition of growth of androgen-dependent LNCaP and castration-resistant C4-2 human prostate cancer cells by AR/p52 inhibitors

**Figure 3:**
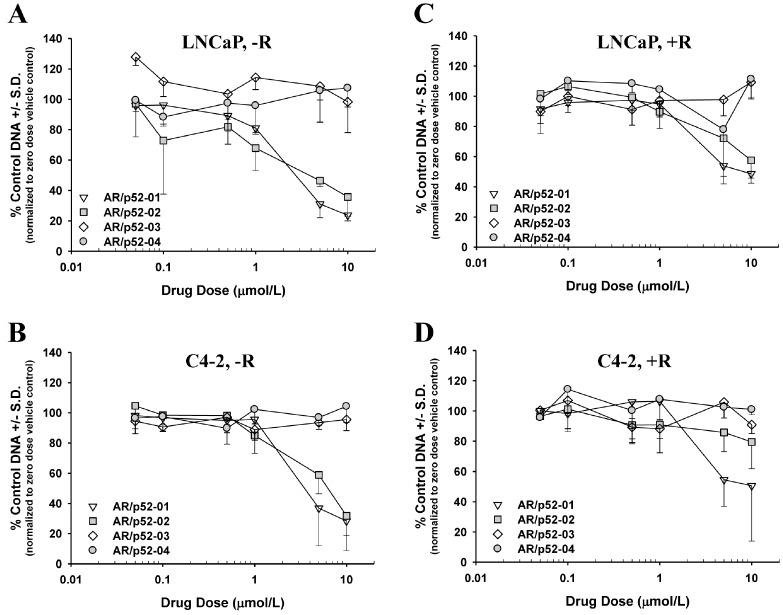
Screening of compounds for inhibition of LNCaP and C4-2 cell growth Compounds were tested for their ability to inhibit growth of LNCaP cells (A,C) or C4-2 cells (B,D) in low androgen medium (−R) (A,B) or medium containing physiologic level 1nM synthetic androgen R1881 (+R) (C,D). Cells were treated with incremental doses of compound or zero dose compound vehicle control for 96h. Dose response effects on growth compared to zero dose control (% control DNA) are shown. Growth IC_50_ Table 1. N=6 to 12 per data point.

Since it has been shown that aberrant activation of AR as a result of its interaction with p52 causes the castration-resistant growth of prostate cancer cells in an androgen-deprived environment [10], we studied the effect of the AR/p52 inhibitors on growth of parental androgen-dependent LNCaP and its castration-resistant variant C4-2 human prostate cancer cells under normal physiologic androgen levels (1 to 2 nM synthetic androgen R1881, denoted by +R) [33] compared to very low androgen levels (denoted by −R) conditions. Dose response growth curves were determined for each compound following 96 hours of treatment with the four selected compounds under the −R and +R conditions in both cell lines using our published assay [32,33] and are shown in Figure 3. The dose at which growth was inhibited by 50% compared to control (IC_50_) was determined from each growth curve and the data are summarized in Table 1. Compounds AR/p52-01 and AR/p52-02 showed significant inhibition of growth in both cell lines under the –R condition (Figures 3A, 3B), with IC_50_ values of ∼3 and 4μM in LNCaP and 4 and 6μM in C4-2 cells, respectively. AR/p52-01 and AR/p52-02 showed some growth inhibition under the +R condition (Figures 3C, 3D), but to a much lesser degree, with IC_50_ values of ∼8μM or greater. Compounds AR/p52-03 and AR/p52-04 at concentrations up to 10 μmol/L had no effect on growth of LNCaP or C4-2 cells under either of the –R or +R conditions and therefore were not explored further.

### Reduction of AR transcriptional activity by inhibitor AR/p52-02 in androgen-dependent LNCaP cells and castration-resistant C4-2 cells

**Figure 4:**
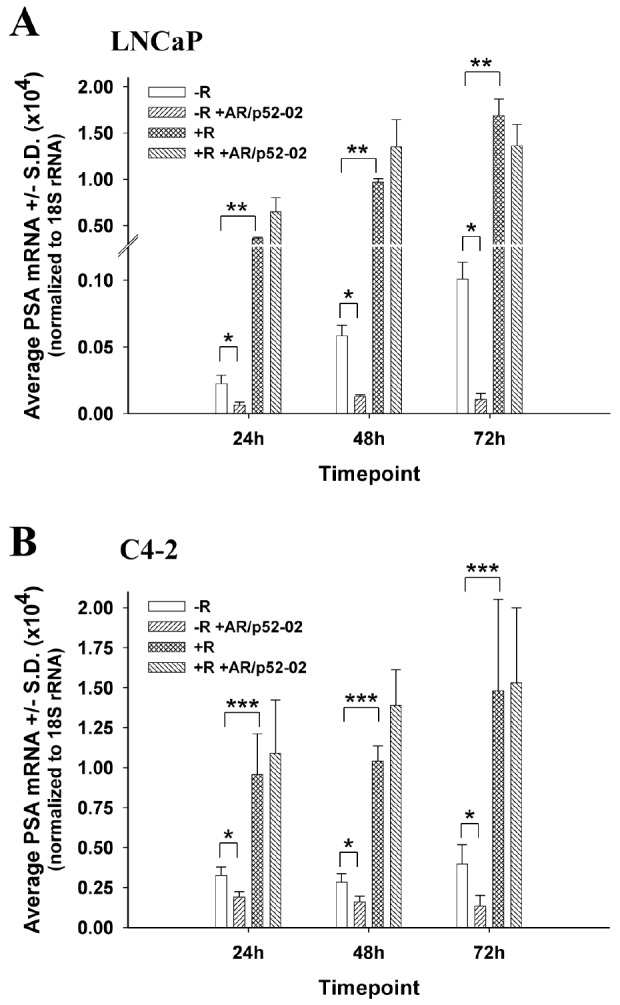
AR/p52-02 decreases PSA mRNA under low androgen conditions in LNCaP and C4-2 cells LNCaP (A) or C4-2 (B) cells were grown overnight in low androgen-medium, then treated with or without 10 μM AR/p52-02 in the presence or absence (±) of 2nM synthetic androgen R1881 (R). After 24, 48 or 72h, the cells were collected using Invitrogen's Cells-to-cDNA kit for quantitative real time PCR analysis to evaluate the level of expression of PSA under each condition. AR/p52-02 significantly reduced AR transcriptional activity as measured by PSA expression in LNCaP and C4-2 cells under low androgen (−R) condition but did not affect androgen induction (+R) of AR transcriptional activity: AR/p52-02 significantly reduced PSA mRNA under low androgen condition (* *P*<0.05 for −R compared to –R+AR/p52-02 at all timepoints) in both LNCaP and C4-2 cells. As expected, 2nM androgen stimulation led to a significant ∼16-fold increase in PSA mRNA (**P<0.001 for +R compared to –R at all timepoints) for LNCaP cells. C4-2 cells also responded to androgen stimulation, but with only ∼3-fold increase (***P<0.01 for all comparisons). AR/p52-02 did not affect PSA mRNA levels under the 2nM androgen-stimulated condition in either cell line, as no difference was observed at any timepoint for +R compared to +R+AR/p52-02. N=6 per condition between two experiments.

PSA expression is a marker of AR transcriptional activity [10]. To determine the effect of the growth inhibitory AR/p52 inhibitors on AR transcriptional activity, the level of PSA mRNA expression was determined over time under –R and +R conditions. As shown in Figures 4A and 4B, AR/p52-02 significantly reduced AR transcriptional activity as measured by PSA mRNA expression in LNCaP and C4-2 cells under –R condition, but did not affect the androgen-induced (+R) increase in AR transcriptional activity. Average PSA mRNA was significantly lower (*P*<0.05) in AR/p52-02 treated cells compared to control for both cell lines in –R at 24h, 48h or 72h of treatment. Of note, AR transcriptional activity, as measured by PSA mRNA, in the –R condition was markedly greater in C4-2 cells compared to LNCaP cells at all timepoints (note the difference in Y-axes in Figures 4A and 4B), and remained steady in C4-2 versus increased in LNCaP over time: C4-2 average PSA mRNA was 15-fold higher at 24h, 5-fold higher at 48h and 4-fold higher at 72h compared to LNCaP in –R (Figures 4A, B). Interestingly, PSA mRNA was reduced by AR/p52-02 to approximately the same level at each timepoint for each cell line in –R. Stimulation with 2nM androgen (+R) led to a significant (P<0.001) ∼16-fold increase in PSA mRNA compared to –R condition at all timepoints in LNCaP cells as expected (Figure 4A). C4-2 cells also responded to androgen stimulation (Figure 4B), but more mildly with only ∼3-fold increase over the higher baseline level of PSA in C4-2 cells (P<0.01 for all comparisons). AR/p52-02 did not affect PSA mRNA levels under 2nM androgen stimulation, as no difference was observed in AR/p52-02 treated cells compared to control at any time point under the +R condition in LNCaP (Figure 4A) or C4-2 (Figure 4B) cells. AR transcriptional activity as measured by PSA mRNA levels was not reduced by AR/p52-01 treatment in C4-2 cells in both –R and +R conditions (data not shown), thus the compound was set aside for further studies on mechanism of action in the future.

### Inhibitor AR/p52-02 does not notably reduce AR nuclear translocation, but does markedly reduce p52 nuclear translocation in androgen-dependent LNCaP cells

**Figure 5:**
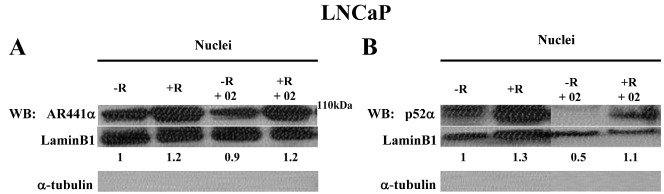
Effect of AR/p52-02 on levels of AR and p52 NF-κB subunit in androgen-dependent LNCaP cells LNCaP cells were grown overnight in low androgen medium, then treated with or without 5 μM AR/p52-02 (02) in the presence or absence (±) of 2nM synthetic androgen R1881 (R) for 72h. After treatment the nuclear protein extracts were separated and the levels of AR and p52 were assessed by western blotting (WB). As expected, androgen increased the nuclear AR (A) and p52 (B) levels (+R compared to –R) in LNCaP cells. The nuclear AR levels were not substantially affected by AR/p52-02 treatment compared to respective –R and +R untreated controls (A). In contrast, nuclear levels of p52 were substantially reduced by treatment with AR/p52-02 compared to respective controls under both the –R and +R conditions (B). LaminB1 was used as loading control. The ratio of AR/LaminB1 is shown below each lane, with the –R control ratio set to 1 in each blot. Lack of α-tubulin showed nuclear purity. Experiments were repeated at least three times.

Since the transcriptional activity of AR and p52 is related to their nuclear levels, the effect of AR/p52-02 on nuclear AR and p52 levels was determined. Nuclear protein extracts from LNCaP cells treated with 5μM of AR/p52-02 inhibitor for 72h or untreated (control) LNCaP cells under the –R and +R conditions were analyzed by western blot for levels of AR (Figure 5A) and p52 (Figure 5B). As expected, stimulation with androgen (+R) led to an increase in nuclear levels of AR (Figure 5A) and p52 (Figure 5B) compared to the low androgen (–R) condition. Treatment with AR/p52-02 did not markedly affect the nuclear level of AR under either –R or +R conditions (Figure 5A, –R compared to –R+02, +R compared to +R+02). In contrast, an abrogation of p52 nuclear translocation due to AR/p52-02 treatment was observed under low androgen (−R) condition (Figure 5B, −R compared to –R+02), and a marked decrease in nuclear p52 level in the presence of androgen was observed for AR/p52-02 treated cells when compared to the control (Figure 5B, +R compared to +R+02).

### Inhibitor AR/p52-02 reduces nuclear translocation of both AR and p52 under low androgen condition in castration-resistant C4-2 cells

**Figure 6:**
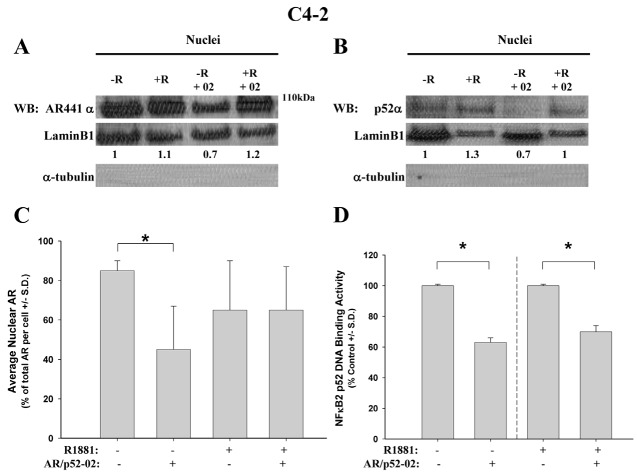
Effect of AR/p52-02 on levels of AR and p52 NF-κB subunit in castration-resistant C4-2 cells C4-2 cells were grown overnight in low androgen medium, then treated with or without 10 μM AR/p52-02 (02) in the presence or absence (±) of 2nM synthetic androgen R1881 (R) for 72h, and analyzed by western blot (WB) for nuclear AR (A) and p52 (B). Nuclear levels were further examined by immunocytochemistry (ICC) for AR nuclear levels (C) and by TransAM™ assay for p52 DNA binding activity as indicator of nuclear p52 levels (D). Western blot indicated that AR/p52-02 caused a reduction in nuclear levels of AR under the –R (low androgen) condition (A), substantiated by ICC results showing significant reduction of nuclear AR by drug under the –R condition (C). Both western (B) and binding activity (D) assays showed that treatment with AR/p52-02 caused a significant decrease in nuclear levels of p52 under both –R and +R conditions. For WB, LaminB1 was used as loading control, and the ratio of AR/LaminB1 is shown below each lane, with the –R control ratio set to 1 in each blot; lack of α-tubulin showed nuclear purity; and experiments were repeated three times. Per condition N ≥ 9 for AR ICC assay and N=3 for p52 DNA binding assay. Dashed line signifies that TransAm™ assays of –R and +R samples were run separately. * *P*<0.05 compared to respective –R or +R untreated control.

The effect of AR/p52-02 on nuclear levels of AR and p52 in the castration-resistant C4-2 cells was similarly determined. Nuclear protein extracts from C4-2 cells treated with 10μM of AR/p52-02 for 72h under the –R and +R conditions were analyzed by western blot for levels of AR (Figure 6A) and p52 (Figure 6B). Similar to LNCaP cells, stimulation with androgen (+R) led to an increase in nuclear p52 in C4-2 cells (Figure 6B, –R compared to +R). The data from western blot also showed a marked reduction in nuclear p52 level by treatment with AR/p52-02 in C4-2 cells under both –R and +R conditions (Figure 6B, −R compared to −R+02, +R compared to +R+02). Analysis of nuclear protein extracts from similarly treated C4-2 cells for p52 DNA binding activity substantiated these results: binding of p52 to its DNA consensus sequence in the nuclear extracts, indicating nuclear level / binding activity of p52, was significantly reduced (P<0.05) to 60% of control under –R condition and to 70% of control under +R condition in C4-2 cells treated with AR/p52-02 compared to respective –R and +R untreated controls (Figure 6D). In contrast to LNCaP cells, stimulation with androgen did not lead to an increase in AR nuclear levels in C4-2 cells (Figure 6A –R compared to +R without AR/p52-02), and treatment with AR/p52-02 markedly reduced AR nuclear level under the –R condition (Figure 6A, –R compared to –R+02), but not under the +R condition (Figure 6A, +R compared to +R+02). Immunocytochemistry analysis of nuclear levels of AR in C4-2 cells treated similarly substantiated these data: average nuclear AR level was significantly decreased (*P*<0.05) by ∼2-fold in C4-2 cells treated with AR/p52-02 under the –R condition, while no difference was observed under the +R condition (Figure 6C).

### Inhibitor AR/p52-02 reduces the phosphorylation of AR at serine81 (pARser81) in low androgen condition

**Figure 7:**
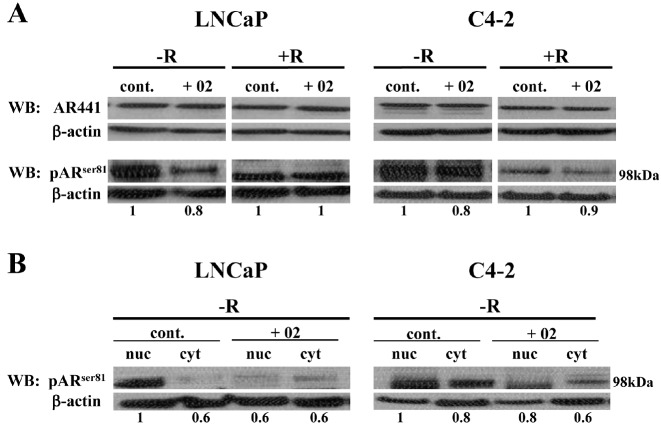
Effect of AR/p52-02 on AR phosphorylation Inhibitor AR/p52-02 reduces the ser81 phosphorylation of androgen receptor (AR) in LNCaP and C4-2 cells in low androgen condition. LNCaP and C4-2 cells were treated with or without 5 μM or 10 μM AR/p52-02 (02), respectively, in the presence or absence (±) of 2nM synthetic androgen R1881 (R) for 72h and the levels of total phosphorylated AR^ser81^ (pAR^ser81^) and AR (AR441) were analyzed by western blotting (WB) in whole cell lysates (A). Levels of pAR^ser81^ in nuclei (nuc) versus cytoplasm (cyt) were also assessed by western blotting (B). Total AR levels were not changed with the treatment as no change was observed under any conditions (A). Total pAR^ser81^ levels were reduced by AR/p52-02 in both LNCaP and C4-2 cells under the low androgen condition (−R), but no effect was seen in the presence of androgen (+R) (A). Nuclear levels in LNCaP cells were decreased by AR/p52-02, while in C4-2 cells both nuclear and cytoplasmic levels were decreased under the –R condition (B). No effect of AR/p52-02 on nuclear or cytoplasmic levels was seen under the +R condition (data not shown). β-actin was used as loading control, and ratios of pAR^ser81^/β-actin are shown below each lane, with the ratio for control in each blot set to 1. Experiments were repeated three times.

Since it has been reported that phosphorylation of AR at the serine81 site (pAR^ser81^) is required for AR stabilization, nuclear localization and transactivation [3-8], we determined the effect of AR/p52-02 on levels of pAR^ser81^. As shown in Figure 7A, western blot analysis of whole cell lysates from LNCaP and C4-2 cells that were treated with 5μM and 10μM of AR/p52-02, respectively, under –R or +R conditions for 72h showed a marked reduction in pAR^ser81^ levels under low androgen (–R) condition in cells treated with AR/p52-02 when compared to –R control for both cell lines, however no differences were observed for AR/p52-02 treated cells compared to untreated cells under the physiologic androgen (+R) condition. The level of total AR remained unchanged under the same conditions (Figure 7A). Subsequent western blot analysis of nuclear and cytoplasmic fractions from similarly treated LNCaP and C4-2 cells showed that nuclear translocation of pAR^ser81^ was reduced by treatment with AR/p52-02 compared to corresponding controls under the –R condition in both cell lines (Figure 7B), while no differences in nuclear and cytoplasmic levels of pAR^ser81^ were observed under the +R condition (data not shown). Interestingly, AR/p52-02 also reduced cytoplasmic levels of pAR^ser81^ in C4-2 cells, but not in LNCaP, under the –R condition

### Inhibitor AR/p52-02 induces ubiquitination of the AR, thereby reducing AR stability

**Figure 8:**
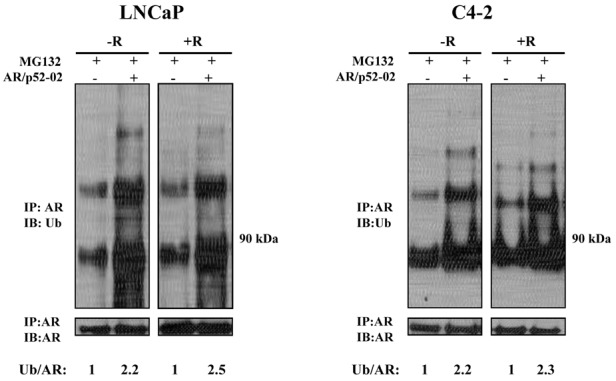
Effect of AR/p52-02 on stability of AR Inhibitor AR/p52-02 causes the instability of AR by increasing its ubiquitination and proteasomal degradation. LNCaP and C4-2 cells were treated with proteasome inhibitor MG132 with (+) or without (−) AR/p52-02 inhibitor for 12h in the presence (+) or absence (−) of 2nM androgen R1881 (R). The cell lysates were subjected to immunoprecipitation using AR441 antibody (IP:AR), and the immunoprecipitates were analyzed by immunoblotting using the antibodies against ubiquitin (Ub) and AR. Increased AR ubiquitination, was observed in LNCaP and C4-2 cells treated with AR/p52-02 in both –R and +R conditions. The ratios of ubiquinated AR from IP:AR,IB:Ub per total AR from IP:AR,IB:AR are shown below each lane, with the ratio for the control in each blot set to 1. Experiments were repeated three times.

To determine whether AR/p52-02 affects AR stability, we investigated the effect of the inhibitor on the degradation of AR by the ubiquitination-proteasome pathway, which is well investigated and is one of the predominant mechanisms of AR degradation [7]. Cell lysates from LNCaP and C4-2 cells that were treated for 12h with proteasome inhibitor MG132 (5μM) in the presence or absence of 5μM and 10μM of AR/p52-02, respectively, under the –R and +R conditions were subjected to immunoprecipitation with AR antibody and analyzed by immunoblotting using antibody against ubiquitin (Figure 8). A substantial increase in ubiquitination of AR was observed under both –R and +R conditions in both cell lines in the presence of AR/p52-02 compared to respective untreated controls when the proteasomal machinery was inhibited by MG132 (Figure 8). Thus AR/p52-02 caused an increase in ubiquitination of AR, which likely led to reduced stability of AR in both LNCaP and C4-2 cells under both –R and +R conditions.

### AR/p52-02 inhibitor decreases p21 in the presence of androgen

**Figure 9:**
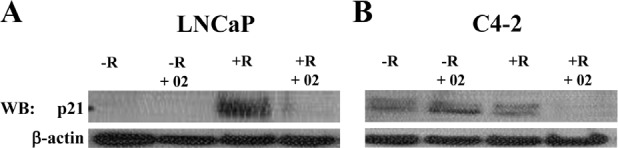
AR/p52-02 reduces the level of p21 in the presence of androgen The level of p21 in whole cell lysate of LNCaP and C4-2 cells that were treated with 5μM and 10μM of AR/p52-02 (02) inhibitor, respectively, in the presence or absence (±) of 2nM synthetic androgen R1881 (R) for 72 h was determined by western blotting (WB) using antibody for p21. In the absence of R1881 (−R) in the control or in AR/p52-02 treated LNCaP cells (A) p21 was not detectable, however in the presence of androgen an induction of p21 was observed (+R compared to –R) that was diminished with AR/p52-02 (+R+02). In C4-2 cells (B) p21 was detected at similar levels in –R and +R, and was decreased by AR/p52-02 treatment under the +R condition. β-actin was used as protein loading control. Experiments were repeated three times.

In a previous study, we showed that this compound significantly reduced cyclinD1 levels in LNCaP and C4-2 cells in the presence of androgen [32]. While this could be due to changes in p52, which has been reported to induce induce the expression of cyclinD1 [23], it could also be due to p21^(WAF-1/CIP1)^ protein, which can be regulated by AR [29] and is known to regulate cyclinD1 activity [34]. Thus, we investigated the effect of AR/p52-02 on p21^WAF/CIP1^ levels in LNCaP and C4-2 cells treated for 72 h with or without AR/p52-02 under the –R and +R conditions. Western blot analysis of whole cell lysates showed significantly higher p21 expression in the +R condition compared to –R condition in LNCaP cells, that was largely reduced by treatment with 5μM of AR/p52-02 under +R (Figure 9A). No expression of p21^(WAF-1/CIP1)^ under –R condition in control untreated LNCaP cells was detected and this did not change with AR/p52-02 treatment (Figure 9A). In contrast, p21^(WAF-1/CIP1)^ was detected at similarly low levels in +R and –R conditions in C4-2 cells (Figure 9B, +R compared to –R), and interestingly was decreased by AR/p52-02 treatment under the +R condition but not under the –R condition (Figure 9B).

## DISCUSSION

Prostate cancer cells rely on AR in all stages of growth and progression [35]. Anti-androgens are commonly used as a component of androgen deprivation therapy (ADT) to prevent AR from binding to its activating ligand, androgen. Earlier generation anti-androgens, e.g., bicalutamide and flutamide, were initially successful in treating prostate cancer patients. However, resistance to therapy started to emerge, and continues even for the next-generation anti-androgens, e.g., enzalutamide, which is administered post bicalutamide. Different mechanisms pertaining to aberrant activation of AR in castration-resistant conditions that do not involve binding of androgens to AR have been suggested for developing resistance to both previous and recent generation anti-androgens [10, 36]. Studies have shown that a novel constitutively active AR splice variant lacking most of the LBD domain mediates prostate cancer anti-androgen therapy resistance [37]. Also, blocking AR activation by a small molecule that binds to the N-terminal domain (NTD) can inhibit castration-resistant prostate cancer cell growth [38]. Furthermore, other transcription factors may activate AR in the absence of androgen [10,35]. One such transcription factor is the non-canonical p52 NF-κB subunit protein [10]. Aberrant activation of AR by p52 has been suggested as a mechanism of growth of human prostate cancer cells and resistance to enzalutamide under the castration condition [10, 13, 18]. Therefore, agents that block the interaction of AR and p52 could potentially prevent the progression and/or inhibit the growth of castration-resistant prostate cancer.

Here, we identified two specific inhibitors of AR/p52 interaction, AR/p52-01 and AR/p52-02, that are capable of inhibiting the growth of both parental androgen-dependent LNCaP and its castration-resistant C4-2 variant human prostate carcinoma cell lines under androgen-deprived conditions with IC_50_ values less than 10 μM. Although the IC_50_ of AR/p52-01 was lower than that for AR/p52-02, the latter reduced the AR transcriptional activity under low androgen conditions more efficiently, as shown by the observed reduction in PSA expression by AR/p52-02 compared to no effect on PSA by AR/p52-01. Therefore, we selected AR/p52-02 for further mechanism of action studies. Interestingly, AR/p52-02 was also previously found to inhibit the interaction between AR and JunD in our study on an ROS generation pathway in prostate cancer cells [32]. A possible explanation of this finding is that spatial conformation of AR in regard to interacting with other co-activator(s) only exposes certain portion(s) of its structure to other proteins, and since we determined that AR/p52-02 does not bind to the ligand binding domain (LBD) of AR, which is consistent with our prior study [32], we may conclude that AR/p52-02 binds to either the N-terminal domain (NTD) or DNA binding domain (DBD) of AR, or interferes at the interface between AR and p52 NF-κB subunit and/or JunD.

To decipher the mode of action of AR/p52-02, we investigated its effect on AR and p52 individually under low androgen condition at growth inhibitory (near IC ) concentrations, which we showed to be non-toxic to the cells in our previous study noted above [32]. AR/p52-02 did *not* affect AR nuclear translocation in the androgen-dependent LNCaP cells, but nuclear translocation of p52 was dramatically decreased. While total nuclear AR was not changed by AR/p52-02 treatment after 72h, the level of nuclear phosphorylated AR^ser81^ (pAR^ser81^) was reduced, and that, along with the reduction of nuclear p52, may explain the significant reduction in PSA expression under the low androgen condition in LNCaP cells.

As for the castration-resistant C4-2 cells, both AR and p52 nuclear translocations were diminished by AR/p52-02 under low androgen condition. AR and p52 are both constitutively expressed in castration-resistant C4-2 cells [10,18], and it has been shown that overexpression of p52 in the the parental LNCaP cells leads to activation of AR and induction of PSA in the absence of androgen [10]. Thus the higher basal level of PSA in C4-2 cells compared to LNCaP cells that we observed may well be related to the higher expression of both AR and p52, and their interaction / binding to the PSA promoter under low androgen(−R) conditions. Therefore the inhibition of the nuclear translocation, and thereby activity, of both transcription factors, AR and p52, by AR/p52-02 may lead to a significant decrease in PSA expression, as was expected and observed in this study. It is to be noted that total AR and p52 (data not shown) were not changed by treatment with AR/p52-02 in either cell line.

We also studied the effect of AR/p52-02 under physiologic androgen levels, i.e., 1 to 2 nM synthetic androgen R1881, in comparison to the low androgen condition. In the presence of 1 nM R1881 (growth inhibitory concentration of androgen [33]), the IC_50_ of AR/p52-02 is higher than under the low androgen condition for both LNCaP and C4-2 cells, greater than 10μM dose range. One possible explanation is that at this concentration of androgen, cells are almost in growth *arrest* and therefore will only respond to higher concentration of the compound. This is supported by our present study, as no significant change in nuclear AR in the *presence* of androgen by AR/p52-02 treatment compared to the corresponding controls was observed in either cell line. However, nuclear level of p52 was reduced in AR/p52-02 treated LNCaP cells in R1881-supplemented condition (+R) as well as in the absence of R1881(−R), when compared to the corresponding controls. Furthermore, marked reduction in nuclear p52 levels under androgen-induced condition in C4-2 cells was observed as well. From these data, we may conclude that activation of AR by androgen predominates over alternative activation pathway(s), such as its interaction with p52, and therefore blocking AR/p52 interaction by the inhibitor in the presence of androgen *does not* cause significant change(s) in the activation of AR that has already been activated by androgen, even though it may inhibit p52 nuclear translocation. This is in accordance with *1)* the growth data that indicates the requirement of higher IC (greater than 10μM dose) for both the parental androgen-dependent LNCaP and castration-resistant C4-2 variant cells in the presence of growth inhibitory concentration of R1881 (e.g., 1-2nM), and *2)* that the level of PSA expression was *not* significantly affected by AR/p52-02 treatment under this androgen-stimulated condition. In our future studies we will investigate the mechanism of action of AR/p52-02 at higher doses in LNCaP and C4-2 cells in the *presence* of androgen.

The importance of ser81 phosphorylation for AR stabilization, nuclear translocation and transcriptional activity under androgen deprivation has been shown [39,40]. Functionally, phosphorylation at AR ser81 promotes cell growth [41]. The significant reduction of nuclear pAR^ser81^ level under low androgen condition in both LNCaP and C4-2 by AR/p52-02 at growth inhibitory concentrations further supports that phosphorylation of AR at ser81 is important for growth of prostate cancer cells under low androgen condition. It may also suggest that pAR^ser81^ is important for the interaction of p52 with AR, consistent with reports that activation of AR by phosphorylation can also act as a means of cross-talk with other signaling pathways, specifically under low androgen conditions which is relevant to castration-resistant prostate cancer [40]. Additionally, since ser81 is located at the N-terminal domain (NTD) of AR, these results may suggest that inhibitor AR/p52-02 binds to the NTD of AR, which could be the region of interaction of AR and p52 as well. Alternatively or in addition, the abrogation of nuclear translocation of p52 by AR/p52-02 inhibitor could be due to masking of the Nuclear Localization Sequence (NLS) of p52 [42] by this inhibitor, which could be the point of interaction of p52 with AR. Furthermore, the decrease in AR stability, as evidenced by increased AR ubiquitination may be the result of reduction in AR phosphorylation by AR/p52-02, since phosphorylation increases AR stability [40]. Based on our observation, the level of ubiquitination of AR in both cell lines was equally affected by AR/p52-02 inhibitor in low androgen or androgen supplemented conditions. Presumably, the outcome of AR/p52-02 action may have caused an overall instability of AR. pAR^ser81^ is only one of many different forms of AR as there are other modifications that are important for AR stability and activity [43], which may have been affected by this inhibitor. In our future studies we will investigate specifically the effect of AR/p52-02 inhibitor on the ubiquitination of pAR^ser81^. Future studies will also focus on the specific mechanism(s) by which AR/p52-02 blocks the interaction of AR and p52. Regardless of the exact mechanism by which AR/p52-02 interferes, the reduction of pAR^ser81^ nuclear translocation and decreased stability of AR caused by the inhibitor likely were major factors in the decrease in AR transcriptional activity and associated inhibition of growth of the prostate cancer cells under the androgen deprived condition. In addition, inhibition of nuclear translocation of p52 and its interaction with AR by AR/p52-02 likely led to a decrease in AR transcriptional activity (as shown by a decrease in PSA mRNA) and growth inhibition of PCa cells under low androgen condition, which would be consistent with the findings of Nadiminty *et. al*, where p52 was found to activate AR via interaction at the NTD of AR, causing increased AR transcriptional activity and increased growth of PCa cells under androgen deprivation [10].

In our previous studies on this compound for inhibition of AR-JunD interaction, we showed that repression of cyclinD1 may be a factor in the growth inhibitory effect of the compound [32]. Since it has been shown that *direct* up-regulation of cyclinD1 expression by p52 leads to the proliferation of tumor cells [23], we propose that the cyclinD1 repression by AR/p52-02 may be due to the reduction of translocation of p52 to the nucleus that we show in the present study. As previously stated [32], the effect of this inhibitor on cyclinD1 was more pronounced under high ROS condition in AR/JunD oxidative stress generation pathway. In our recent publication [19], we also hypothesized that there is a connection between ROS generation pathway involving AR/JunD interaction and feed-forward loop involving p52. These data support the possibility that the diminished presence of p52 in the nuclei of AR/p52-02 treated cells is responsible, at least in part, for the decrease in cyclinD1.

It is very intriguing that AR/p52-02 inhibitor represses both p21^WAF1/CIP1^ and cyclinD1, as this is in contrast with the role of p21, which is typically known as a proapoptotic/growth inhibitory protein and more importantly as a down regulator of cyclinD1 [23]. However, there are studies that have shown p21^WAF/CIP1^ is not always a growth inhibitory protein and rather point to the different role of p21^WAF/CIP1^ as a proliferative and mitogenic protein in prostate cancer progression [24-28, 34]. Furthermore, it has also been shown that p21 ^WAF/CIP1^ regulates cyclinD1 activity by assembling CDK4/cyclinD and CDK6/cyclinD complexes, and in this role p21^WAF/CIP1^ actually protects cyclinD1 from cytoplasmic degradation [34]. It was also reported that p21^WAF1/CIP1^ is an androgen/AR target gene and is induced by androgen [29], as we also have shown here in the LNCaP cells where we observed significant induction of p21 by androgen treament. A connection between androgen/AR/p21^WAF1/CIP1^/cyclinD1 has been reported and this connection is proposed to be important for prostate cancer progression [44]. The same study also reported that p21^WAF1/CIP1^ levels are frequently associated with a more proliferative and predominantly nuclear cyclin D1 phenotype. Unlike LNCaP cells, in C4-2 cells a low basal level of p21^WAF/CIP1^ was observed under all conditions and was not markedly changed by androgen induction, which may indicate an alternative pathway for p21 expression in C4-2 cells. However, in both LNCaP and C4-2 cells, AR/p52-02 diminishes p21^WAF/CIP1^ in the presence of androgen. This is consistent with our previous study [32] in which the decrease in cyclinD1 levels by this compound was more pronounced in the presence of androgen. The compound may disrupt the androgen/AR signaling axis that affects p21^WAF1/CIP1^ expression, downstream of androgen activation of AR, which may supersede other pathways affecting p21^WAF1/CIP1^. It is plausible to think that inhibitor AR/p52-02 interrupts the nuclear localization and activity of pAR^ser81^ and subsequently diminishes the level/activity of p21^WAF1/CIP1^ and cyclinD1, and concurrently abrogates nuclear translocation of p52 that further blocks expression of cyclinD1 by the p52 pathway. However, further study of these pathways and other pathways regulating p21^WAF1/CIP1^ and cyclinD1 are needed to better understand the signaling networks between these factors. Overall, the study revealed some interesting differences between androgen-dependent LNCaP and its castration-resistant variant C4-2 cells that will be important to further investigate, particulary in relation to the effect of AR/p52 inhibitors on AR and p52 activation and regulation of cell growth.

The specificity of AR/p52-02 inhibitor of AR/p52 interaction was further examined by assessing the level of other proteins in canonical and non-canonical NF-κB pathways eg, p65/p50, IκB-α and β and IKKα/β. No significant change in the level of protein expression of these factors has been observed by AR/p52-02 inhibitor (data not shown). We believe that the small molecule inhibitor of the interaction of AR and p52 NF-κB subunit, AR/p52-02, represses the castration-resistant prostate cancer cell growth by blocking both AR and p52 pathways and may thereby prevent the transition of androgen-dependent growth of prostate cancer cells to castration-resistant growth. Studies to further delineate the mechanism of action of AR/p52-02 and/or analogues may help further the understanding of how AR and p52 are important in castration-resistant prostate cancer and potentially unearth additional pathway(s) targets for development of therapies for prostate cancer.

## MATERIALS AND METHODS

### Cell culture

Androgen-dependent LNCaP human prostate carcinoma cells were obtained from the American Type Culture Collection. Castration-resistant LNCaP variant C4-2 cells were a kind gift from Ajit Verma (Department of Human Oncology, UW-Madison), with permission from George Thalmann (Department of Urology, Inselspital, Bern, Switzerland). Cells are routinely tested for mycoplasma using MycoAlert® Mycoplasma Detection Kit (Lonza, Rockland, ME) approximately every six months. The cell lines were authenticated by short tandem repeat DNA profiling, as well as verified to be free of mycoplasma, via DDC Medical (Fairfield, OH) in December 2013. LNCaP cells were maintained in Dulbecco's Modified Eagle Medium (DMEM; gibco® by Life Technologies, cat.#31600-034) supplemented with 5% fetal bovine serum (FBS; gibco® by Life Technologies, cat.# 16000-044) and 1% antibiotic/antimycotic, and seeded in DMEM supplemented with 1% FBS and 4% charcoal-stripped serum (F1C4) and 1% antibiotics for studies. The F1C4 combination of charcoal stripped and non-stripped serum was shown before to sufficiently deplete androgen content while limiting the adverse growth effects not related to hormone depletion that occur with the use of 5% stripped serum [45]. C4-2 cells were maintained and seeded for studies in DMEM F1C4. For studies, LNCaP and C4-2 cells were collected via trypsinization with 0.05% Trypsin-EDTA (gibco® by Life Technologies, cat.#25300-054) and seeded as follows: for growth assays, cells were seeded at 4000 cells/well in 96-well culture plates; for protein and RNA analyses, cells were seeded at a density of ∼4×104 cells / cm2 in various sizes of culture plates; and for immunocytochemistry analyses cells were seeded at 20,000 cells/well in Corning® BioCoat™ poly-d-lysine 8-well glass culture slides (Discovery Labware, Inc., Bedford, MA; Corning cat.# 354632). Cells were grown under low androgen level (F1C4) condition, which is estimated to be lower than androgen levels in serum of castrated male patients [45], or under normal physiologic androgen levels with addition of 1 to 2 nM synthetic androgen R1881 (methyltrienolone, NEN) as described before [33]. For AR ubiquitination studies, proteasome inhibitor MG132 (cat.#C2211) from Sigma (St. Louis, MO) was used at 5 μM to inhibit proteasome formation. Treatments with R1881, AR/p52 inhibitors and MG132 or respective vehicle controls were initiated three days after seeding for growth studies or one day after seeding for all other studies, and carried out for the designated timepoints at 37°C under 5% CO_2_. Inhibitor AR/p52-02 was used at a dose of 5 μM in LNCaP cells versus 10 μM in C4-2 cells due to an overall stronger inhibition of growth of LNCaP cells in 72h mode of action studies.

Hep3B human hepatoma cells with no endogenous AR were obtained from the Small Molecule Screening and Synthesis Facility at the University of Wisconsin Carbone Cancer Center (UWCCC SMSSF) and maintained and seeded for transfection studies in RPMI 1640 supplemented with 10% FBS and 1% antibiotics.

### Antibodies used for western blots and ICC

NF-κB2 p100/p52 rabbit monoclonal, human specific (18D10) was purchased from Cell Signaling Technology (Danvers, MA). Mouse monoclonal antibody for AR (AR441; sc-7305) was obtained from Santa Cruz Biotechnology (Santa Cruz, CA). Rabbit monoclonal antibody for pAR^ser81^ (04-078) was obtained from Millipore (Billerica, MA). Ubiquitin rabbit polyclonal antibody (ab7780) for detection of Ubiquitinated-AR was obtained from Abcam (Cambridge, MA). For controls for purity of cytoplasmic and nuclear extracts, mouse monoclonal α-tubulin (DM1A) (cat#CP06) from Calbiochem (San Diego, CA) and rabbit polyclonal antibody for LaminB1 (ab16048) from Abcam (Cambridge, MA) were used, respectively. For detection of fusion protein p52-Gluc2, rabbit polyclonal antibody against *Gaussia* Luciferase (#401) was obtained from Nanolight Technology (Pinetop, AZ). For detection of p21, mouse monoclonal antibody against p21 from Santa Cruz Biotechnology (sc-817) was used. For loading control, β-actin mouse monoclonal antibody (A5441) was obtained from Sigma (St. Louis, MO). HRP conjugated secondary antibodies; goat anti-mouse (31430) from Pierce Biotechnology (Thermo Fisher, Waltham, MA) and donkey anti-rabbit (NA934) from Amersham™ (by GE Healthcare, Pittsburgh, PA) were used. For immunocytochemistry, secondary antibody Alexa Fluor 488 donkey anti-mouse (A21202) from Life Technologies (Grand Island, NY) was used.

### Vector construction

cDNA for p52 NF-κB subunit in CMV4-vector [pCMV4-p52(human)] that was kindly provided by Shannon Kenney at the University of Wisconsin-Madison was amplified by PCR and subcloned into pcDNA3.1(+) (Life Technologies, Grand Island, NY). This construct was used for fusing the C-terminal portion of *Gaussia* Luciferase (Gluc2) [20] at the end of the p52 gene after removing the stop codon. The construct was named cmv-p52-Gluc2. An out of frame control vector was also constructed, cmv-OFp52-Gluc2. The construction of cmv-Gluc1-AR fusion vector was reported before [31].

### Transfection of constructs into Hep3B cells and bioluminescence activity of reconstituted *Gaussia* Luciferase

Hep3B cells (5×10^5^) were seeded and 1 day later co-transfected with 3μg each of cmv-Gluc1-AR and cmv-p52-Gluc2 vectors or transfected with cmv-Gluc1-AR and cmv-OFp52-Gluc2 as a negative control using Lipofectamine 2000 reagent (Invitrogen) following the manufacturer-supplied protocol. Two to 3 hours after transfection, cells were washed and refed with DMEM without serum. Cells were collected 48h later and lysed for measurement of *Gaussia* Luciferase (GL) activity using Biolux Gaussia assay kit (E3300L) from New Engalnd Biolabs (Ipswich, MA) per the manufacturer's protocol.

### High throughput screening for specific inhibitors of AR/p52 interaction using large scale lysates from Hep3B cells co-transfected with cmv-Gluc1-AR and cmv-p52-Gluc2

Large scale cell lysates from Hep3B cells that were transfected and harvested as described above were generated for use in a high throughput screen (HTS) based on reconstituted GL bioluminescence. Screening of 2,800 small molecules from a Life Chemicals Library for inhibitors of AR interaction with p52 NF-κB subunit was carried out as described before for AR-JunD interaction [32], with the exception that in the present study, the overnight incubation of plates containing lysate with potential small molecule inhibitors of AR/p52 was carried out at room temperature instead of 37°C.

For eliminating false positives, including non-specific inhibitors and toxins, a secondary screen was performed on the hits identified from the initial HTS, using lysate from cells that were co-transfected with vectors containing cmv-Gluc1/cmv-Gluc2 (negative control) or with cmv-smad3-Gluc1/cmv-Gluc2-PKB (positive control) [20], essentially as described before [32] with the exception that the overnight incubation was done at room temperature. Only the hits that inhibited GL reconstitution by greater than 25% in the AR/p52 system but failed to inhibit reconstitution of GL with positive control in the secondary screen were considered as “Confirmed Hits”. The detailed procedure for HTS and confirmation of hits was reported before [32]. For all screens, our Z'-factor was above 0.56.

### Fluorescence polarization AR ligand binding competition assay

A commercially available assay, PolarScreen™ Androgen Receptor Competitor Assay kit (Invitrogen), was used to determine if the AR/p52 inhibitors identified by the HTS had anti-androgenic (i.e., AR-LBD binding) activity. Fluorescence polarization assay with graded concentrations of the compounds of interest was performed according to the manufacturer's supplied protocol, in comparison to clinical anti-androgen Casodex®(bicalutamide).

### Growth assay

For growth assay, DNA levels of control and treated cell cultures were measured as an indicator of growth [33]. The detailed procedure was published before [32]. Briefly, cells in 96-well culture plates were treated with graded concentrations of compounds, with or without 1nM R1881. After 96h, plates were washed with Kreb's Ringer buffer, fresh buffer was added and the plates were frozen. Subsequently plates were thawed, incubated with Hoechst dye at 6.7μg/mL in high salt TNE buffer, and scanned on a plate reader using 360nm excitation / 460nm emission. Hoechst-DNA fluorescence units represented cell number in the micro-culture plates for a measure of cell culture growth, per our standard protocol [46].

### PSA cDNA synthesis and quantitative real time pcr (qrtpcr)

Cells treated with or without 10μM AR/p52-02 in the presence or absence of 2nM R1881 were harvested for qrtpcr analysis to determine PSA mRNA levels after 72h of treatment. For qrtpcr, cells-to-cDNA™ II reverse transcription without RNA isolation kit from Ambion^®^ by Life Technologies ™ (AM 1723) was used to synthesize cDNA directly from cells following the instructions provided by the manufacturer as reported before [32]. The cDNAs were subjected to real time PCR using IQ™ Syber^®^Green Supermix (Bio-Rad). Each reaction was normalized by coamplification of 18S rRNA. Triplicates of samples were run on a Bio-Rad CFX-96 real time cycler. The sequences of primers used for amplification of PSA and 18S rRNA cDNA were as follows: PSA: Forward 5′-GACCACCTGCTACGCCTCA and Reverse 5′-GGAGGTCCACACTGAAGTTTC; 18S rRNA: Forward 5′-CGCCGCTAGAGGTGAAATCT and Reverse: 5′-CGAACCTCCGACTTTCGTT.

### Western blots and immunoprecipitation/immunoblot

For protein analyses, cells were treated for 72h in the presence or absence of 2nM R1881 with or without inhibitor AR/p52-02 at 5μM for LNCaP cells or 10μM for C4-2 cells. Whole cell lysates were prepared using modified RadioImmunoPrecipitation Assay (RIPA) buffer containing a tablet of complete protease inhibitors from Roche (Indianapolis, IN) as reported before [31]. For nuclear and cytoplasmic fractionation, the protocol reported by Liu *et. al* was used [47]. Briefly, cells were lysed by incubation in a low salt buffer containing; 10 mmol/L HEPES-KOH (pH7.9), 1.5 mmol/L MgCl_2_, 10 mmol/L KCl, and 0.1% NP40 supplemented with protease inhibitors (Roche, Basel, Switzerland) and incubated on ice for 30 minutes. Nuclei were precipitated by centrifugation at 3000 *g* for 10 minutes at 4°C. The supernatants were collected as the cytosolic fraction. After washing once with low-salt buffer, nuclei were lysed in high salt lysis buffer containing; 50 mmol/L Tris-HCl (pH8), 150 mmol/L NaCl, 1% Triton X-100 followed by mechanical disruption at 4°C for 30 minutes. The nuclear lysate was cleared by 10,000 rpm centrifugation at 4°C for 15 minutes. Protein concentration was determined using BCA assay kit (Pierce, Rockford, IL). Western blots were performed as described before [32]. Briefly, proteins were separated on 4-12% Bis-Tris gels (NuPAGE Novex, Life Technologies). Gels were transferred onto PVDF membrane, blocked and then incubated with antibody. Membranes were developed with Pierce® ECL (Thermo Scientific) with appropriate HRP-conjugated secondary antibodies.

For AR ubiquitination studies, prior to immunoblot, total protein was extracted, quantified, and 500μl (500μg total protein) was subjected to preclearance using 50 μl Protein A/G PLUS-agarose beads (Sant Cruz Biotechnology,sc-2003) for 20 minutes at room temperature with rotation. The precleared lysates were incubated with 6 μg AR antibody (AR441; Santa Cruz Biotechnology) for an overnight with constant rotation at 4°C. The antibody-protein complexes were pulled down using 50 μl Portein A/G PLUS-agarose beads in an overnight incubation at 4°C with constant rotation following which the beads were separated by centrifugation at 14,000 × g for 1 minute at 4°C. The beads were further washed three times with PBS after which they were boiled with 4 × LDS sample buffer (Novex, Life Technologies). The protein was resolved using SDS-PAGE as explained above and the western blots were probed with Ubiquitin antibody for detection of Ubiquitinated-AR.

### Immunocytochemistry (ICC)

Cells treated with or without inhibitor AR/p52-02 at 5μM for LNCaP cells or 10μM for C4-2 cells in the presence or absence of 2nM R1881 for 72h were prepared for ICC analysis. At the end of treatment, cells were fixed using fixative solution containing 3.6% paraformaldehyde, 0.024% Saponin and 1mM sodium Orthovanadate for 10 minutes at room temperature. The fixation was quenched using 0.1% sodium borohydride in PBS followed by 2 changes of rinse solution I ( Ca^2+^/Mg^2+^-free PBS, 1mM Orthovanadate and 0.012% Saponin). One drop of Image-iTFX signal enhance solution (Invitrogen) was added to each well and incubated for 30 minutes and then washed twice with rinse solution I. The cells were blocked by rinse solution II (1% BSA and 0.3 M Glycine) for an hour at 37°C in a 5% CO_2_ incubator followed by 2-3 times wash with rinse solution I. Three microliter of mouse monoclonal AR antibody (AR441,sc-7305) per well diluted (50x) in rinse solution II was added and incubated for 2 hours at 37°C in a CO_2_ incubator. The cells were Molecule Screening and Synthesis Facility for assistance rinsed and diluted (100x) secondary antibody Alexa Fluor (488) donkey anti-mouse (Invitrogen, A21202) was added to each well. Finally, the slides were mounted using DAPI containing mounting solution. Images were taken at 20x magnification with a Nikon TI-U inverted fluorescence microscope for quantitation of AR fluorescence normalized to DAPI for AR nuclear levels.

### NF-κB2/p52 DNA binding assay

LNCaP and C4-2 cells were treated for 72h with or without 5μM or 10μM of inhibitor AR/p52-02, respectively, in the presence or absence of 2nM R1881. Using TransAM™ NFκB p65/p50/p52 kit from Active Motif (cat#48196; NF-κB2/p52), the binding activity of p52 to its consensus sequence (5′-gggactttcc-3′) with its specific antibody (provided in the kit) was determined in all nuclear fractions of cells from different conditions following the manufacturer's instructions. Briefly, for DNA binding assay, 20μl of nuclear extract containing total nuclear protein of 5μg was added to each well of three wells in a 96-well plate provided in the kit. Four wells were also used for positive control: Raji nuclear extract (provided in the kit) at 20μl/well at different concentrations (e.g., 2μg, 5μg and 7μg). Three wells also were used for negative control: nuclear extraction buffer at 20μl/well. Thirty microliters of complete binding buffer were added to each well and the plate was covered using the provided adhesive film. The mixture of nuclear extract and binding buffer was incubated for 1h at room temperature with mild agitation followed by washing x3 with wash buffer. One hundred microliters of diluted NF-κB2/p52 antibody were added to each well and incubated for 1h at room temperature followed by washing the wells x3 with wash buffer. One hundred microliters of diluted HRP-conjugated secondary antibody were added to each well and incubated for another hour at room temperature followed by washing x4 with wash buffer. After the final wash 100μl of Developing Solution was added to each well and incubated for 5 minutes. The reaction was stopped by adding 100 μl Stop Solution. The absorbance was measured at 450nm.

### Statistical analyses

An unpaired two-tailed heteroscedastic Student's *t*-test with a confidence level of 0.05 was performed for data comparisons and significance determinations.
